# Listeriosis during Pregnancy: A Public Health Concern

**DOI:** 10.1155/2013/851712

**Published:** 2013-09-26

**Authors:** Teresa Mateus, Joana Silva, Rui L. Maia, Paula Teixeira

**Affiliations:** ^1^Centro de Biotecnologia e Química Fina (CBQF) Laboratório Associado, Escola Superior de Biotecnologia, Universidade Católica Portuguesa/Porto, Rua Dr. António Bernardino Almeida, 4200-072 Porto, Portugal; ^2^Departamento de Medicina Veterinária (EUVG), Escola Universitária Vasco da Gama, Coimbra, Portugal; ^3^Faculdade de Ciências Humanas e Sociais—Universidade Fernando Pessoa, Porto, Portugal

## Abstract

*Listeria* was first described in 1926 by Murray, Webb, and Swann, who discovered it while investigating an epidemic infection among laboratory rabbits and guinea pigs. The role of *Listeria monocytogenes* as a foodborne pathogen was definitively recognized during the 1980s. This recognition was the consequence of a number of epidemic human outbreaks due to the consumption of contaminated foods, in Canada, in the USA and in Europe. Listeriosis is especially severe in immunocompromised individuals such as pregnant women. The disease has a low incidence of infection, although this is undeniably increasing, with a high fatality rate amongst those infected. In pregnant women listeriosis may cause abortion, fetal death, or neonatal morbidity in the form of septicemia and meningitis. Improved education concerning the disease, its transmission, and prevention measures for immunocompromised individuals and pregnant women has been identified as a pressing need.

## 1. Introduction

*Listeria monocytogenes* is a Gram positive, facultative intracellular, foodborne pathogen responsible for cases and outbreaks of listeriosis.* Listeria *was first described in 1926 by Murray et al. who discovered it while investigating an epidemic infection among laboratory rabbits and guinea pigs [1]. At that time, it was given the name *Bacterium monocytogenes *because infection in the animals was characterized by monocytosis. The following year, Pirie isolated an identical bacterium from the liver of several gerbils and proposed the name* Listerella* for the genus in honor of Lord Lister a prominent surgeon of the time [2]. Despite considerable confusion in the nomenclature of the pathogen until 1940, the official name *Listeria monocytogenes* was adopted in the Sixth Edition of Bergey's Manual of Determinative Bacteriology [3].

The first cases of human listeriosis were reported by Nyfeldt in 1929 [4]. The increased number of reported cases during the 1980s in several countries, and the evidence of foodborne transmission, turned listeriosis into a recognized foodborne disease [5, 6]. Listeriosis has emerged as an atypical foodborne illness of major public health concern because of the severity of the disease (meningitis, septicemia, and abortion), the high case fatality rate (20–30% of cases), the long incubation period, and the predilection for individuals who have an underlying condition which leads to impairment of T-cell-mediated immunity [7]. In addition, the ubiquitous nature of *L. monocytogenes* and its ability to survive and grow in harsh conditions (wide pH range, high salt concentration, refrigeration temperatures) makes this pathogen of high concern to the food industry.

It has been reported that the annual incidence of listeriosis ranges between 1 and 10 per million population [8]. Although listeriosis is less common than other foodborne diseases, 19% and 17% of the known causes of foodborne disease-related deaths occurring in the USA and France, respectively, are caused by *L. monocytogenes* [9, 10]. 

Most authors comment that 99% of the human *Listeria* infections are from foodborne origin [11–14]. *Listeria monocytogenes* has been recovered from several foods such as meat, milk, and fish products; ice cream; vegetables; and several ready-to-eat foods [15–17]. There are recent surveys of *L. monocytogenes* in foods that demonstrate the presence of this bacterium in several of these foods and ready-to-eat products [12, 17–23]. Also, there have been several listeriosis outbreaks worldwide [24–28], and certain foods have been described as “high risk” for listeriosis. In recent years the annual number of reported listeriosis cases has increased in several European countries [29–33]. Goulet et al. [30] and Shetty et al. [6] suggest that this increase is particularly due to the increasing population aged more than 60 years, or less than 60 years, although with an immunocompromised predisposing condition.

*Listeria monocytogenes* is responsible for cases and outbreaks of febrile gastrointestinal disease in otherwise healthy people and invasive listeriosis, which more usually affects pregnant, newborns, the elderly, and immunecompromised individuals. According to Warriner and Namvar [25] noninvasive listeriosis is associated with a huge intake of *L. monocytogenes*. 

*Listeria monocytogenes* has been differentiated into 13 serotypes; serotypes 1/2a, 1/2b, and 4b have been involved in the majority of reported human listeriosis cases [33]. 

Subtype characterization of *L. monocytogenes* isolates from listeriosis outbreaks has suggested that many outbreaks were caused by a small number of L. monocytogenes epidemic clones [34], that is, by a closely related group of isolates that evolved clonally. Epidemic clones of *L. monocytogenes* have been implicated in several outbreaks and sporadic cases of listeriosis worldwide [35–37], and for this reason, although not yet proven, they have been considered more virulent than other strains.

## 2. High Risk Foods for *Listeria* Infection

High-risk foods have all of the following properties: (1) have the potential for contamination with *L. monocytogenes*  (2) support the growth of *L. monocytogenes *to high numbers, (3) do not require further cooking at home (4) require refrigeration, (5) have an extended shelf life, and (6) are contaminated with high levels of the pathogen at the time of consumption [13, 38, 39]. Ready-to-eat seafood such as smoked fish or mussels, raw seafood including sushi and sashimi, premixed raw vegetables including coleslaw, meat products such as sausages, paté, and rillettes, hot dogs, ham, salami, chicken wraps and deli turkey breast, unpasteurized milk, soft-serve ice creams, and soft cheeses are examples of foods that have been associated with transmission of listeriosis. 

The minimum number of cells capable of causing disease is not known although epidemiological data suggest that it is high (ca. 10^6^ CFU/g) [40]; the infectious dose depends on the strain virulence and the host susceptibility [40, 41]. 

 The characteristics of the disease (the hugely variable and potentially very long incubation periods, the low attack rates, and the rarity of identification of specific food vehicles), provide limited data for calculation of dose responses [42]. 

## 3. Mechanisms of Infection

*Listeria monocytogenes* has the ability to induce its own entry into host cells, usually mammalian cells (human and animal), such as macrophages, epithelial cells, and endothelial cells of the gastrointestinal tract [43]. After ingestion, those cells surviving the low pH in the stomach pass through to the small intestine—the first site where invasion occurs—disseminate from the mesenteric lymph nodes to the spleen and the liver and from there *L. monocytogenes* can reach the brain or the placenta [44], causing, respectively, infections of the central nervous system (CNS) mostly in immunocompromised patients and intrauterine/cervical infections in pregnant women [40].

*Listeria monocytogenes* uses various proteins, including some internalins to adhere and to invade the host cells. Once in the intracellular phagocytic vacuole, bacteria secrete listeriolysins and phospholipases that allow it to lyse the vacuolar membrane and avoid intracellular killing. Upon being released into the cytoplasm, *L. monocytogenes* can multiply and induce the formation of actin filaments which will allow it to move in the cytoplasm until it reaches the plasma membrane. Subsequently, adjacent cells are invaded through plasma membrane protrusions and cell-to-cell spread. Through this cycle *L. monocytogenes* can move from one host cell to another cell, without being in the extracellular environment, thus escaping from the human T-cell immune system, and invading other tissues and organs [45, 46].

## 4. Incubation Period for Listeriosis

Unlike other foodborne diseases, the incubation period for listeriosis can be long. Goulet et al. [47] recently reported an overall median incubation period of invasive listeriosis of 8 days, ranging from 1 to 67 days. For pregnancy-associated cases the authors reported a longer incubation period (median 27.5 days, ranging from 17 to 67 days) than for CNS cases (median 9 days, ranging from 1 to 14 days) and for bacteraemia cases (median 2 days, ranging 1 to 12 days). For febrile gastrointestinal disease, the median incubation period was reported as 24 hours, ranging from 6 hours to 10 days [47].

## 5. Individuals at Higher Risk of Listeriosis

Although exposure and colonization may occur in any person, those patients without predisposing factors represent less than 20% of the cases [48]. Silk et al. [49] reported an incidence rate of listeriosis of 0.27 cases per 100,000 general US population. There are well-defined risk groups for human invasive listeriosis: pregnant women and their fetuses or neonates, the elderly, and cellular immunocompromised individuals such as those who are receiving corticosteroids or chemotherapy, hemodialysis, transplants, diabetic, HIV carriers, and drug dependents [40, 50]. Ogunmodede et al. [51] found that pregnant women have an infection risk twenty times higher than other healthy adults. Incidence rates among pregnant women and adults aged more than 65 years were reported in the USA as 3.42 and 1.21 cases per 100,000 population, respectively [49]. Pregnant Hispanic women are also recognized as having a much higher infection risk than pregnant women of other ethnicities [52, 53], probably due to the habit of the consuming soft cheese [13, 54]. In a recent study by Pouillot et al. [53] it was demonstrated that a significant increase in listeriosis incidence begins among persons as young as 45 years and that incidence rates subsequently increase steadily with age. 

## 6. Invasive Listeriosis

In adults, the most common clinical form of listeriosis is meningitis, due to the bacterial tropism to the central nervous system [44, 55]. Salamano et al. [54] reported that meningitis caused by *Listeria* represents 5% to 11% of all acute bacterial meningitis in adults, reaching 14% in patients aged more than 45 years. Apparently, individuals with predisposing factors seem to suffer more from septicemia rather than meningitis, but this situation may be due to the followup of these individuals by the health system, making them more available for blood collection and culture in the case of a febrile episode [41]. A survey of 782 listeriosis cases reported in 20 countries, showed that 43% of the infections were related with pregnancy, 29% with septicemia, 24% with central nervous system infection, and 4% were atypical forms of the infection [56]. *Listeria *infections may produce sequelae, but the incidence is rarely determined. Up to 11% of neonates and 30% of patients that survive central nervous system infections, suffer from residual symptoms and psychiatric sequelae [52, 56, 57]. Salamano et al. [54] reported a high level of recurrences (7%). 

### 6.1. Listeriosis during Pregnancy

During pregnancy, cellular immunity is minimal due to the increased progesterone [58, 59], making pregnant women particularly susceptible to intracellular microorganisms like *L. monocytogenes *[51, 60]. The vertical cell-to-cell transmission is frequent since *L. monocytogenes* shows uterus and placenta tropism [48, 51]. Immunological aspects of *Listeria* infection during pregnancy were reviewed by Poulsen and Czuprynski [61].

 Goulet et al. [30] reported that the incidence in pregnant women and neonates appears to be decreasing in Europe. On the contrary Sisó et al. [62] reported an increase in the incidence of listeriosis between 2000 and 2010 in a tertiary referral hospital in Barcelona. It is important to point out that listeriosis is not often diagnosed in pregnancy, due to the microbiological diagnostic techniques being difficult, and also due to the histological alterations in the placenta being similar to other diseases, hindering, therefore, a definitive assessment of the importance of listeriosis in pregnancy health [63]. Moreover, pregnant women may be asymptomatic or present nonspecific clinical symptoms (e.g., flu-like symptoms, backache, headache, vomiting/diarrhea, muscle pains, and sore throat) [64–66]. Gupta et al. [67] stated that microbiological examinations are not usually made on spontaneous or premature abortions; thus, it is difficult to infer the actual incidence of listeriosis [68].

Infection by *L. monocytogenes* during pregnancy may result in serious outcomes including miscarriage, stillbirth, chorioamnionitis, preterm delivery, and maternal and neonatal sepsis [65, 66].

It is agreed that there is an increased susceptibility for listeriosis in late pregnancy. A third of the listeriosis cases affect pregnant women mostly in the third trimester of pregnancy [48, 58, 59], rarely in the second and only exceptionally occurs in the first trimester [63]. Studies by Sisó et al. [62] reported an incidence of the infection two times higher after 28 weeks of gestation than in the first trimesters. Listeriosis during early gestations generally has a poorer prognosis for fetuses as opposed to later gestations and commonly results in miscarriage or stillbirth [62, 65, 67]. DiMaio [48] commented that iron supplement, commonly prescribed during pregnancy, favors the development of listeriosis. 

A transplacentary fetal infection may result in abortion, as already stated, premature birth, or an infected neonate [54, 68]. Laciar et al. [68] Doyle [58] and Schwab and Edelweiss [69] agree that most cases of the intrauterine infection are a consequence of the transplacentary dissemination after bacteremia in pregnant women and less frequently due to an ascending contamination from the pregnant woman's lower genital tract colonized by *L. monocytogenes, *as the vagina and cervix are also potentially carrier sites of this microorganism [48]. More commonly, infection during the passage of the fetus through the birth canal may result in the nosocomial transmission in the neonatal nursery [70]. Two distinct infection forms are described in the newborns. 

#### 6.1.1. Early-Onset Neonatal Listeriosis

This is acquired *in utero*, by transplacentary transmission, and is also known as granulomatosis infantiseptica. Disease in pregnancy precedes the fetal infection, although symptoms may not be specific [48, 70]. This infection, generally occurring at a mean of 36 h after birth [71], and probably due to aspiration of infected amniotic liquid, is characterized by clinical features like septicemia (81–88%), respiratory distress or pneumonia (38%), and meningitis (24%) [72, 73]. The formation and dissemination of abscesses and granulomas in multiple organs may occur [55]. The mortality rate for infants born alive approaches 20%, and the frequency of abortion and stillbirth increases the overall mortality rate to more than 50% [74].

#### 6.1.2. Late-Onset Neonatal Listeriosis

Infection occurs particularly during delivery, by contamination, with symptoms such as meningitis or meningoencephalitis together with septicemia, occurring 2 or 3 weeks after delivery [70]. In these cases deliveries are not complicated and the transmission may be in the passage through the birth canal; the more frequent occurrence during deliveries by caesarean section being relevant, suggesting nosocomial transmission [48, 52]. 

The mortality rate associated with late-onset disease is ca. 10% [69], but as commented by DeWaal et al. [75] and Buzby [76] a high rate of surviving babies develop severe and chronic neurological complications, for example, delayed mental development and blindness. 

### 6.2. Diagnosis of Listeriosis

The diagnosis of listeriosis depends upon detecting the growth of the microorganisms in corporal fluids normally considered as sterile-blood, amniotic, and cerebrospinal fluids [48, 57]. A rapid diagnosis would be important for preventing recurrences in pregnancies and to treat neonatal infections that may cause meningitis with sequelae such as hydrocephaly, delayed mental development, convulsions, and other neurological symptoms [58, 75, 76]. 

The diagnosis of listeriosis in pregnancy and early treatment would be possible if all the febrile episodes during pregnancy were evaluated through a blood culture [67, 68]. For this purpose, pregnant women should report any disease symptom, because listeriosis can be apparently harmless for the pregnant woman but severely infect the fetus [52, 75, 77]. 

### 6.3. Treatment**  **of**  **Listeriosis

Listeriosis often requires antimicrobial therapy. The treatment of choice consists of a *β*-lactam antibiotic, normally ampicillin, alone or in combination with an aminoglycoside, classically gentamicin. Second-line agents in case of allergy to *β*-lactams or in certain disease states include trimethoprim/sulfamethoxazole, erythromycin, vancomycin, and the fluoroquinolones [78]. Resistance of clinical isolates of *L. monocytogenes* to these antibiotics is low [79]. Early diagnosis and antibiotic administration increases the probability of a favorable outcome [62, 78].

## 7. How to Reduce the Incidence of Listeriosis

Due to the high mortality rate of listeriosis, it is necessary to reduce its incidence. For that purpose, it is urgent to identify the most efficacious strategies based on risk assessment from food production to consumption [13, 48]. 

According to the International Life Sciences Institute [13], the use of scientifically-based messages, to high-risk groups and in health professionals, education is one of the main strategies to adopt concerning the reduction of listeriosis incidence, because it is generally accepted that in some foods, *L. monocytogenes* absence may not be possible. These strategies should minimize food contamination and educate the population in general and risk groups in particular, as well as health professionals and care providers [13, 41]. Cates et al. [80] concluded from their study about listeriosis information transmission to pregnant women that if we want the message to be well delivered to those at risk, it should be targeted and appealing to the pregnant woman; provide detailed information about sources, prevention measures, morbidity, and mortality rates (but as simple as “1 in 4”, rather than 25%); give emphasis to words like “abortion”, “premature birth”; and be transmitted by the obstetrician or other doctor. “Preventative” public alerts about concerning listeriosis prevention are already available (e.g., http://www.cdc.gov/pregnancy/infections-listeria.html; http://ideas.health.vic.gov.au/diseases/listeria-facts.asp; http://americanpregnancy.org/pregnancycomplications/listeria.html; http://www.listeriosisprevention.com/facts.html; http://www.health.state.mn.us/divs/idepc/diseases/listeriosis/index.html). Wong et al. [81] commented that in the USA, doctors are considered by the population as the most credible source of information about health. Doctors are important food safety educators as during the year, they are in contact with 80% of the population, and also because people are more likely to change behaviors if they have been recently ill. Hence, campaigns about food safety education targeted for doctors are recommended [81], especially for obstetricians, as pregnant women will not change their attitudes if they do not recognize the source of the information or if they do not believe it [82]. 
